# subs2vec: Word embeddings from subtitles in 55 languages

**DOI:** 10.3758/s13428-020-01406-3

**Published:** 2020-08-12

**Authors:** Jeroen van Paridon, Bill Thompson

**Affiliations:** 1 Max Planck Institute for Psycholinguistics, Nijmegen, The Netherlands; 2 Princeton University, Princeton, NJ 08544 USA

**Keywords:** Word embeddings, Distributional semantics, Lexical norms, Multilingual

## Abstract

This paper introduces a novel collection of word embeddings, numerical representations of lexical semantics, in 55 languages, trained on a large corpus of pseudo-conversational speech transcriptions from television shows and movies. The embeddings were trained on the OpenSubtitles corpus using the fastText implementation of the skipgram algorithm. Performance comparable with (and in some cases exceeding) embeddings trained on non-conversational (Wikipedia) text is reported on standard benchmark evaluation datasets. A novel evaluation method of particular relevance to psycholinguists is also introduced: prediction of experimental lexical norms in multiple languages. The models, as well as code for reproducing the models and all analyses reported in this paper (implemented as a user-friendly Python package), are freely available at: https://github.com/jvparidon/subs2vec.

## Introduction

Recent progress in applied machine learning has resulted in new methods for efficient induction of high-quality numerical representations of lexical semantics—*word vectors*—directly from text. These models implicitly learn a vector space representation of lexical relationships from co-occurrence statistics embodied in large volumes of naturally occurring text. Vector representations of semantics are of value to the language sciences in numerous ways: as hypotheses about the structure of human semantic representations (e.g. Chen et al., (2017)); as tools to help researchers interpret behavioral (e.g. Pereira et al., (2016)) and neurophysiological data (e.g. Pereira et al., (2018)), and to predict human lexical judgements of e.g., word similarity, analogy, and concreteness (see Methods for more detail); and as models that help researchers gain quantitative traction on large-scale linguistic phenomena, such as semantic typology (e.g. Thompson et al., (2018)), semantic change (e.g. Hamilton et al., (2016)), or linguistic representations of social biases (e.g. Garg et al., (2018)), to give just a few examples.

Progress in these areas is rapid, but nonetheless constrained by the availability of high quality training corpora and evaluation metrics in multiple languages. To meet this need for large, multilingual training corpora, word embeddings are often trained on Wikipedia, sometimes supplemented with other text scraped from web pages. This has produced steady improvements in embedding quality across the many languages in which Wikipedia is available (see e.g. Al-Rfou et al., (2013), Bojanowski et al., (2017), and Grave et al., (2018));^1^ large written corpora meant as repositories of knowledge. This has the benefit that even obscure words and semantic relationships are often relatively well attested.

However, from a psychological perspective, these corpora may not represent the kind of linguistic experience from which people learn a language, raising concerns about psychological validity. The linguistic experience over the lifetime of the average person typically does not include extensive reading of encyclopedias. While word embedding algorithms do not necessarily reflect human learning of lexical semantics in a mechanistic sense, the semantic representations induced by any effective (human or machine) learning process should ultimately reflect the latent semantic structure of the corpus it was learned from.

In many research contexts, a more appropriate training corpus would be one based on conversational data of the sort that represents the majority of daily linguistic experience. However, since transcribing conversational speech is labor-intensive, corpora of real conversation transcripts are generally too small to yield high quality word embeddings. Therefore, instead of actual conversation transcripts, we used television and film subtitles since these are available in large quantities.

That subtitles are a more valid representation of linguistic experience, and thus a better source of distributional statistics, was first suggested by New et al., (2007) who used a subtitle corpus to estimate word frequencies. Such subtitle-derived word frequencies have since been demonstrated to have better predictive validity for human behavior (e.g., lexical decision times) than word frequencies derived from various other sources (e.g. the Google Books corpus and others; Brysbaert and New (2009), Keuleers et al., (2010), and Brysbaert et al., (2011)). The SUBTLEX word frequencies use the same OpenSubtitles corpus used in the present study. Mandera et al., (2017) have previously used this subtitle corpus to train word embeddings in English and Dutch, arguing that the reasons for using subtitle corpora also apply to distributional semantics.

While film and television speech could be considered only pseudo-conversational in that it is often scripted and does not contain many disfluencies and other markers of natural speech, the semantic content of TV and movie subtitles better reflects the semantic content of natural speech than the commonly used corpora of Wikipedia articles or newspaper articles. Additionally, the current volume of television viewing makes it likely that for many people, television viewing represents a plurality or even the majority of their daily linguistic experience. For example, one study of 107 preschoolers found they watched an average of almost 3 h of television per day, and were exposed to an additional 4 h of background television per day (Nathanson et al., 2014).

Ultimately, regardless of whether subtitle-based embeddings outperform embeddings from other corpora on the standard evaluation benchmarks, there is a deeply principled reason to pursue conversational embeddings: The semantic representations learnable from *spoken* language are of independent interest to researchers studying the relationship between language and semantic knowledge (see e.g. Lewis et al., (2019) and Ostarek et al., (2019)).

In this paper we present new, freely available, subtitle-based pretrained word embeddings in 55 languages. These embeddings were trained using the fastText implementation of the skipgram algorithm on language-specific subsets of the OpenSubtitles corpus. We trained these embeddings with two objectives in mind: to make available a set of embeddings trained on transcribed pseudo-conversational language, rather than written language; and to do so in as many languages as possible to facilitate research in less-studied languages. In addition to previously published evaluation datasets, we created and compiled additional resources in an attempt to improve our ability to evaluate embeddings in languages beyond English.

## Method

### Training corpus

To train the word vectors, we used a corpus based on the complete subtitle archive of OpenSubtitles.org, a website that provides free access to subtitles contributed by its users. The OpenSubtitles corpus has been used in prior work to derive word vectors for a more limited set of languages (only English and Dutch; Mandera et al., (2017)). Mandera and colleagues compared skipgram and CBOW algorithms as implemented in word2vec (Mikolov et al., 2013a) and concluded that when parameterized correctly, these methods outperform older, count-based distributional models. In addition to the methodological findings, Mandera and colleagues also demonstrated the general validity of using the OpenSubtitles corpus to train word embeddings that are predictive of behavioral measures. This is consistent with the finding that the word frequencies (another distributional measure) in the OpenSubtitles corpus correlate better with human behavioral measures than frequencies from other corpora (Brysbaert and New, 2009; Keuleers et al., 2010; Brysbaert et al., 2011).

The OpenSubtitles archive contains subtitles in many languages, but not all languages have equal numbers of subtitles available. This is partly due to differences in size between communities in which a language is used and partly due to differences in the prevalence of subtitled media in a community (e.g., English language shows broadcast on Dutch television would often be subtitled, whereas the same shows may often be dubbed in French for French television). While training word vectors on a very small corpus will likely result in impoverished (inaccurate) word representations, it is difficult to quantify the quality of these vectors, because standardized metrics of word vector quality exist for only a few (mostly Western European) languages. We are publishing word vectors for every language we have a training corpus for, regardless of corpus size, alongside explicit mention of corpus size. These corpus sizes should not be taken as a direct measure of quality, but word vectors trained on a small corpus should be treated with caution.

### Preprocessing

We stripped the subtitle and Wikipedia corpora of non-linguistic content such as time-stamps and XML tags. Paragraphs of text were broken into separate lines for each sentence and all punctuation was removed. All languages included in this study are space-delimited, therefore further parsing or tokenization was not performed. The complete training and analysis pipeline is unicode-based, hence non-ASCII characters and diacritical marks were preserved.

After preprocessing, we deduplicated the corpora in order to systematically remove over-represented, duplicate material from the corpus. While Mandera et al., (2017) deduplicated by algorithmically identifying and removing duplicate and near-duplicate subtitle documents, we performed deduplication by identifying and removing duplicate lines across the whole corpus for each language as advocated by Mikolov et al., (2018). This method was used for both the subtitle and Wikipedia corpora. Line-wise deduplication preserves different translations of the same sentence across different versions of subtitles for the same movie, thus preserving informative variation in the training corpus while still removing uninformative duplicates of highly frequent lines such as “Thank you!”.

Finally, bigrams with a high mutual information criterion were transformed into single tokens with an underscore (e.g., ”New York” becomes ”New_York”) in five iterations using the Word2Phrase tool with a decreasing mutual information threshold and a probability of 50% per token on each iteration (Mikolov et al., 2013b).

### fastText skipgram

The word embeddings were trained using fastText, a collection of algorithms for training word embeddings via context prediction. FastText comes with two algorithms, CBOW and skipgram (see Bojanowski et al., (2017), for review). A recent advancement in the CBOW algorithm, using position-dependent weight vectors, appears to yield better embeddings than currently possible with skipgram (Mikolov et al., 2018). No working implementation of CBOW with position-dependent context weight vectors has yet been published. Therefore, our models were trained using the current publicly available state of the art by applying the improvements in fastText parametrization described in Grave et al., (2018) to the default parametrization of fastText skipgram described in Bojanowski et al., (2017); the resulting parameter settings are reported in Table 1.

**Table 1:** fastText skipgram parameter settings used in the present study

Parameter	Value	Description
minCount	5	Min. number of word occurrences
minn	3	Min. length of subword ngram
maxn	6	Min. length of subword ngram
t	.0001	Sampling threshold
lr	.05	Learning rate
lrUpdateRate	100	Rate of updating the learning rate
dim	300	Dimensions
ws	5	Size of the context window
epoch	10	Number of epochs
neg	10	Number of negatives sampled in
		the loss function

### Evaluation of embeddings

A consensus has emerged around evaluating word vectors on two tasks: predicting human semantic similarity ratings and solving word analogies. In the analogies domain, the set of analogies published by Mikolov et al., (2013b) has emerged as a standard and has been translated into French, Polish, and Hindi by Grave et al., (2018) and additionally into German, Italian, and Portuguese (Köper et al., 2015; Berardi et al., 2015; Querido et al., 2017). Semantic similarity ratings are available for many languages and domains (nouns, verbs, common words, rare words) but the most useful for evaluating relative success of word vectors in different languages are similarity sets that have been translated into multiple languages: RG65 in English (Rubenstein and Goodenough, 1965), Dutch (Postma & Vossen, 2014), German (Gurevych, 2005) and French (Joubarne & Inkpen, 2011), MC30 (a subset of RG65) in English (Miller & Charles, 1991), Dutch (Postma & Vossen, 2014), and Arabic, Romanian, and Spanish (Hassan & Mihalcea, 2009), YP130 in English (Yang & Powers, 2006) and German (Meyer & Gurevych, 2012), SimLex999 in English (Hill et al., 2014) and Portuguese (Querido et al., 2017), Stanford Rare Words in English (Luong et al., 2013) and Portuguese (Querido et al., 2017), and WordSim353 in English (Finkelstein et al., 2001), Portuguese (Querido et al., 2017), and Arabic, Romanian, and Spanish (Hassan & Mihalcea, 2009).

Additional similarity datasets we could only obtain in just a single language are MEN3000 (Bruni et al., 2012), MTurk287 (Radinsky et al., 2011), MTurk771 (Halawi et al., 2012), REL122 (Szumlanski et al., 2013), SimVerb3500 (Gerz et al., 2016) and Verb143 (Baker et al., 2014) in English, Schm280 (a subset of WS353; Schmidt et al., (2011)) and ZG222 in German (Zesch and Gurevych, 2006), FinnSim300 in Finnish (Venekoski & Vankka, 2017), and HJ398 in Russian (Panchenko et al., 2016).

#### Solving analogies

To add to the publicly available translations of the so-called Google analogies introduced by Mikolov et al., (2013a), we translated these analogies from English into Dutch, Greek, and Hebrew. Each translation was performed by a native speaker of the target language with native-level English proficiency. Certain categories of syntactic analogies are trivial when translated (e.g., adjective and adverb are identical wordforms in Dutch). These categories were omitted. In the semantic analogies, we omitted analogies related to geographic knowledge (e.g., country and currency, city and state) because many of the words in these analogies are not attested in the OpenSubtitles corpus. Solving of the analogies was performed using the cosine multiplicative method for word vector arithmetic described by Levy and Goldberg (2014) (see (1)).

1 $$ \arg\max_{b^{*} \in V} = \frac{\cos(b^{*},b)\cos(b^{*},a^{*})}{\cos(b^{*},a)+\varepsilon} $$

For analogies of the form *a* is to *a*^∗^ as *b* is to *b*^∗^. With small but non-zero *ε* to prevent division by zero. Equation reproduced here from Levy and Goldberg (2014).

#### Predicting lexical norms

To support experimental work, psycholinguists have collected large sets of *lexical norms*. Brysbaert et al., (2014b), for instance, collected lexical norms of *concreteness* for 40,000 English words, positioning each on a five-point scale from highly abstract to highly concrete. Lexical norms have been collected for English words in a range of semantic dimensions. Significant attention has been paid to *valence, arousal, dominance* (13K words, Warriner et al., (2013)), and *age of acquisition* (30K words, (Kuperman et al., 2012)). Other norm sets characterize highly salient dimensions such as *tabooness* (Janschewitz, 2008). In a similar, but more structured study, Binder et al., (2016) collected ratings for 62 basic conceptual dimensions (e.g., *time, harm, surprise, loud, head, smell*), effectively constructing 62-dimensional psychological word embeddings that have been shown to correlate well with brain activity.

Norms have been collected in other languages too. Although our survey is undoubtedly incomplete, we collated published norm sets for various other, less studied languages (see Tables 2 and 3 for an overview). These data can be used to evaluate the validity of computationally induced word embeddings in multiple languages. Prior work has demonstrated that well-attested lexical norms (i.e., Valence, Arousal, Dominance, and Concreteness in English) can be predicted with reasonable accuracy using a simple linear transformation of word embeddings (Hollis and Westbury, 2016). Using this approach, the lexical norms can be understood as gold-standard unidimensional embeddings with respect to human-interpretable semantic dimensions. In general this relationship has been exploited to use word embeddings to predict lexical norms for words that no norms are available for (e.g. Bestgen and Vincze (2012), Hollis et al., (2017), Recchia and Louwerse (2015a), Recchia and Louwerse (2015b), Turney and Littman (2003), Vankrunkelsven et al., (2015), Westbury et al., (2013), Bestgen (2008), Feng et al., (2011), Turney and Littman (2002), and Dos Santos et al., (2017)), although this procedure should be used with caution, as it can introduce artefacts in a predicted lexical norm, especially for norms that are only weakly predictable from word embeddings (see Mandera et al., (2015), for an extensive discussion of this issue).

**Table 2:** Lexical norms datasets. 1/2

Language	Article	Lexical norms	Number of words	Number of raters
Dutch	Brysbaert et al., (2014a)	Age of acquisition, concreteness	25888	15 per item
Dutch	Keuleers et al., (2015)	Prevalence	52847	300 per item
Dutch	Roest et al., (2018)	Arousal, insulting, taboo (general),	672	87 per item
		taboo (personal), valence		
Dutch	Speed and Majid (2017)	Arousal, auditory, dominance, gustatory,	485	15 per item
		modality exclusivity, olfactory, tactile,		
		valence, visual		
Dutch	Verheyen et al., (2019)	Age of acquisition, arousal, concreteness,	1000	20 per item
		dominance, familiarity, imageability, valence		
English	Brysbaert et al., (2014b)	Concreteness	37058	25 per item
English	Brysbaert et al., (2019)	Prevalence	61855	388 per item
English	Engelthaler and Hills (2018)	Humorousness	4997	35 per item
English	Janschewitz (2008)	Familiarity, offensiveness, tabooness,	460	78 per item
		personal use		
English	Keuleers et al., (2012)	Lexical decision time	28515	39 per item
English	Kuperman et al., (2012)	Age of acquisition	30121	20 per item
English	Lynott et al., (2019)	Lancaster sensorimotor norms	39707	25 per item
English	Pexman et al., (2019)	Body–object interaction	9349	26 per item
English	Scott et al., (2019)	Age of acquisition, arousal, concreteness,	5553	20 per item
		dominance, familiarity, gender association,		
		imageability, semantic size, valence		
English	Warriner et al., (2013)	Arousal, dominance, valence	13915	20 per item
Farsi	Bakhtiar and Weekes (2015)	Age of acquisition, familiarity, imageability	871	40 per item
Finnish	Eilola and Havelka (2010)	Concreteness, emotional charge, familiarity,	210	150 per item
		offensiveness, valence		
Finnish	Söderholm et al., (2013)	Arousal, valence	420	250 per item
French	Bonin et al., (2018)	Arousal, concreteness, context availability,	1659	30 per item
		valence		
French	Chedid et al., (2019b)	Familiarity	3596	20 per item
French	Chedid et al., (2019a)	Auditory perceptual strength, visual	3596	25 per item
		perceptual strength		
French	Desrochers and Thompson (2009)	Imageability	3600	72 per item
French	Ferrand et al., (2010)	Lexical decision time	38840	25 per item
French	Monnier and Syssau (2014)	Arousal, valence	1031	37 per item

**Table 3:** Lexical norms datasets. 2/2

Language	Article	Lexical norms	Number of words	Number of raters
German	Grandy et al., (2020)	Imageability, emotionality (in two age groups)	2592	20 per item
German	Kanske and Kotz (2010)	Arousal, concreteness, valence	1000	64 per item
German	Schauenburg et al., (2015)	Arousal, authority, community, potency,	858	35 per item
		valence		
Indonesian	Sianipar et al., (2016)	Arousal, concreteness, dominance,	1490	70 per item
		predictability, valence		
Italian	Vergallito et al., (2020)	Auditory, gustatory, haptic, lexical decision	1121	57 per item
		time, modality exclusivity, naming time,		
		olfactory, visual		
Malay	Yap et al., (2010)	Lexical decision time	1510	44 per item
Polish	Imbir (2016)	Arousal, concreteness, dominance,	4905	50 per item
		imageability valence		
Portuguese	Cameirão and Vicente (2010)	Age of acquisition	1749	48 per item
Portuguese	Soares et al., (2012)	Arousal, dominance, valence	1034	50 per item
Spanish	Abella and González-Nosti (2019)	Age of acquisition, motor content	4565	25 per item
Spanish	Díez-Álamo et al., (2018)	Color vividness, graspability,	750	26 per item
		pleasant taste, risk of		
		pain, smell intensity, sound intensity,		
		visual motion		
Spanish	Díez-Álamo et al., (2019)	Sensory experience	5500	35 per item
Spanish	Guasch et al., (2016)	Arousal, concreteness, context availability,	1400	20 per item
		familiarity, imageability, valence		
Spanish	Stadthagen-Gonzalez et al., (2017)	Arousal, valence	14031	20 per item
Spanish	Stadthagen-González et al., (2018)	Anger, arousal, disgust, fear, happiness,	10491	20 per item
		sadness, valence		
Turkish	Göz et al., (2017)	Age of acquisition, imagery, concreteness	600	457 per item

Conversely, the same relationship can be used as an evaluation metric for word embeddings by seeing how well new vectors predict lexical norms. Patterns of variation in prediction can also be illuminating: are there semantic norms that are predicted well by vectors trained on one corpus but not another, for example? We examined this question by using L2-penalized regression to predict lexical norms from raw word vectors. Using regularized regression reduces the risk of overfitting for models like the ones used to predict lexical norms here, with a large number of predictors (the 300 dimensions of the word vectors) and relatively few observations. Ideally, the regularization parameter is tuned to the amount of observations for each lexical norm, with stronger regularization for smaller datasets. However, in the interest of comparability and reproducibility, we kept the regularization strength constant. We fit independent regressions to each lexical norm, using fivefold cross validation repeated ten times (with random splits each time). We report the mean correlation between the observed norms and the predictions generated by the regression model, adjusted (penalized) for any words missing from our embeddings. Because of the utility of lexical norm prediction and extension (predicting lexical norms for unattested words), we have included a lexical norm prediction/extension module and usage instructions in the *subs2vec* Python package.

## Results

Results presented in this section juxtapose three models generated by the authors using the same parametrization of the fastText skipgram algorithm: A *wiki* model trained on a corpus of Wikipedia articles, a *subs* model trained on the OpenSubtitles corpus, and a *wiki+subs* model trained on a combination of both corpora. A priori, we expected the models trained on the largest corpus in each language (wiki+subs) to exhibit the best performance. Performance measures are penalized for missing word vectors. For example: If for only 80% of the problems in an evaluation task word vectors were actually available in the subs vectors, but those problems were solved with 100% accuracy, the reported score would be only 80%, rather than 100%. If the wiki vectors on that same task included 100% of the word vectors, but only 90% accuracy was attained, the adjusted scores (80% vs 90%) would reflect that the Wikipedia vectors performed better. (Unpenalized scores are included in Appendix %app:unpenalizedC, for comparison.)

### Semantic dissimilarities

**Fig. 1:**
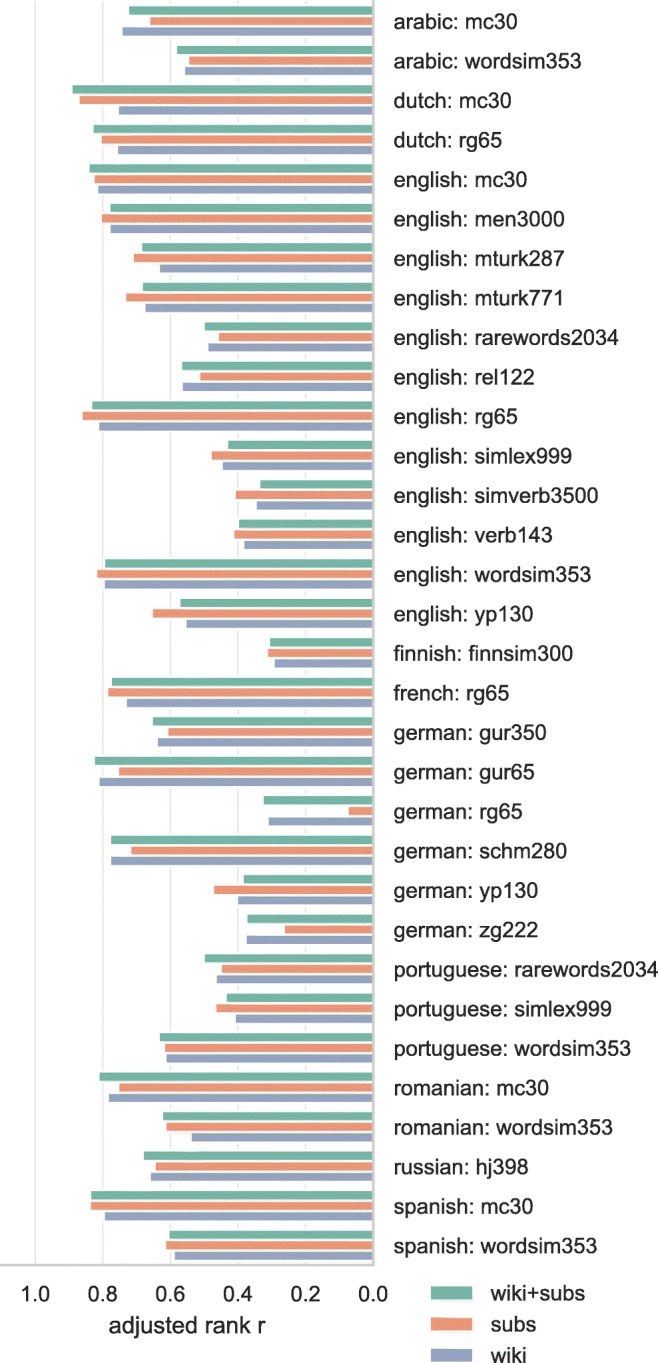
Rank correlations between human ratings of semantic similarity and word vector cosine similarity. Correlations are adjusted by penalizing for missing word vectors

Spearman’s rank correlation between predicted similarity (cosine distance between word vectors) and human-rated similarity is presented in Fig. 1. Performance is largely similar, even for datasets like the Stanford Rare Words dataset where the Wikipedia corpus, by virtue of being an encyclopedia, tends to have more and better training samples for these rare words.

### Semantic and syntactic analogies

**Fig. 2:**
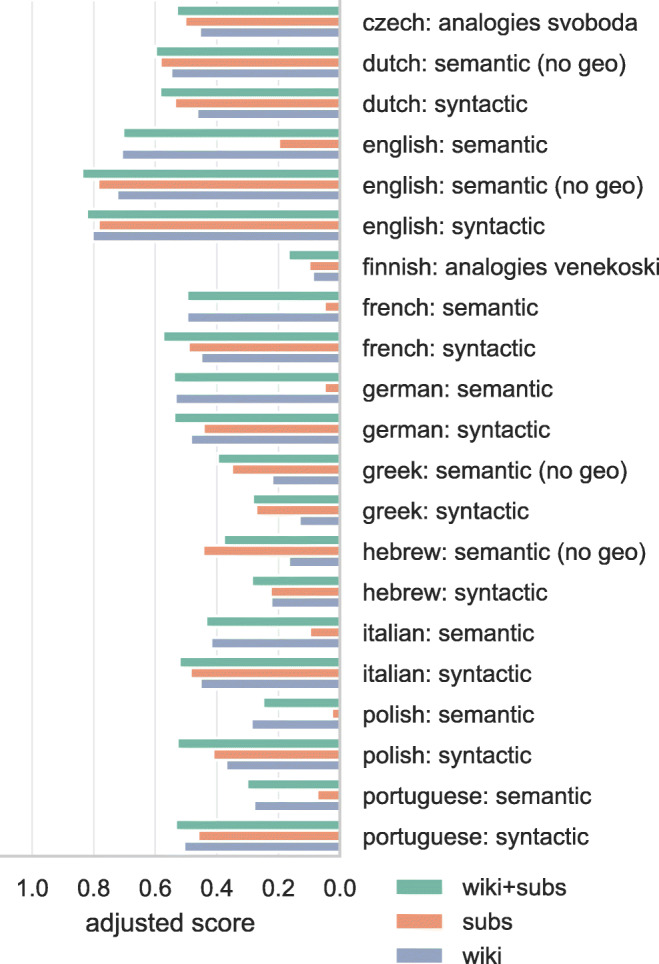
Proportion of correctly solved analogies in the semantic and syntactic domain using word vectors. Semantic datasets contained 93% geographic analogies, *no geo* datasets are those same datasets, excluding the geographic analogies. Scores are adjusted by penalizing for missing word vectors

Adjusted proportion of correctly solved analogies is presented in Fig. 2. Note that while word vectors trained on a Wikipedia corpus strongly outperform the subtitle vectors on the semantic analogies sets, this is mostly due to a quirk of the composition of the semantic analogies: Geographic relationships of the type country-capital, city-state, or country-currency make up 93% of the commonly used semantic analogies. This focus on geographic information suits the Wikipedia-trained vectors, because being an encyclopedia, capturing this type of information is the explicit goal of Wikipedia. However, some of the more obscure analogies in this set (e.g., ”Macedonia” is to ”denar” as ”Armenia” is to ”dram”) seem unlikely to be solvable for the average person (i.e., they do not appear to reflect common world knowledge). In this sense the lower scores obtained with the embeddings trained on the subtitle corpus are perhaps a better reflection of the linguistic experience accumulated by the average person. To better reflect general semantic knowledge, rather than highly specific geographic knowledge, we have removed the geographic analogies in the sets of analogies that were translated into new languages for the present study.

### Lexical norms

**Fig. 3:**
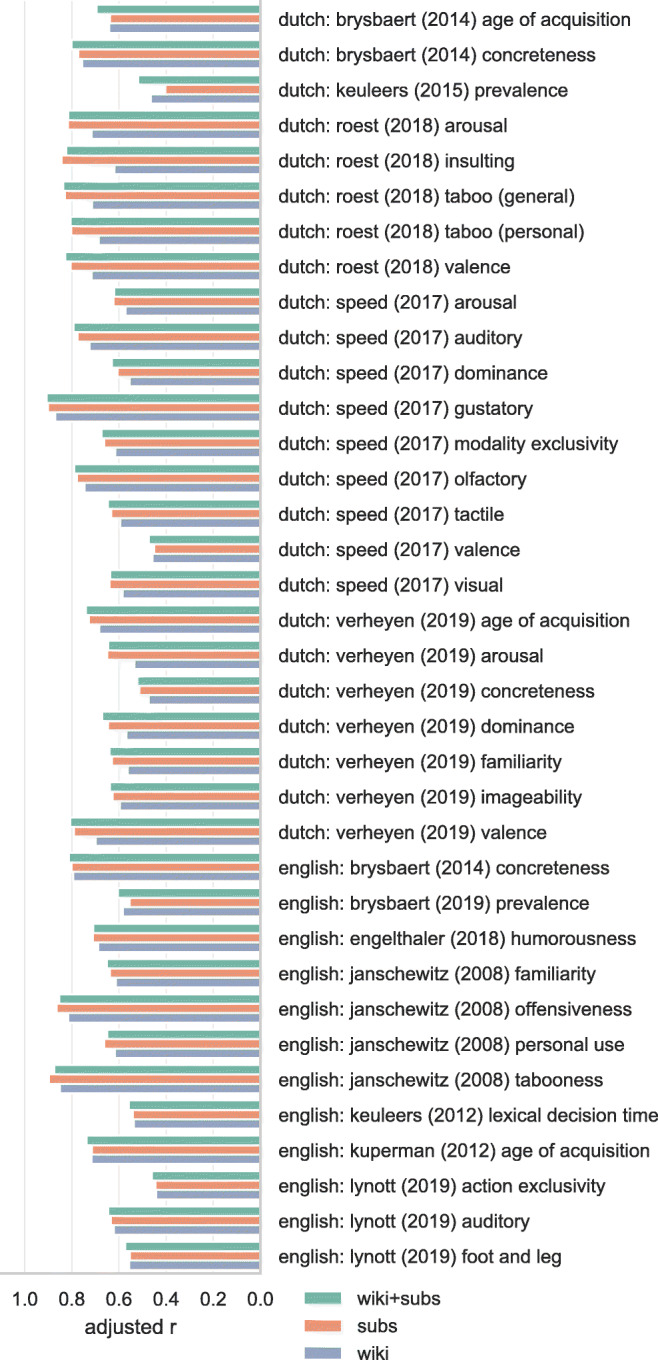
Correlations between lexical norms and our predictions for those norms based on cross-validated ridge regression using word vectors. Correlations are adjusted by penalizing for missing word vectors. 1/4

**Fig. 4:**
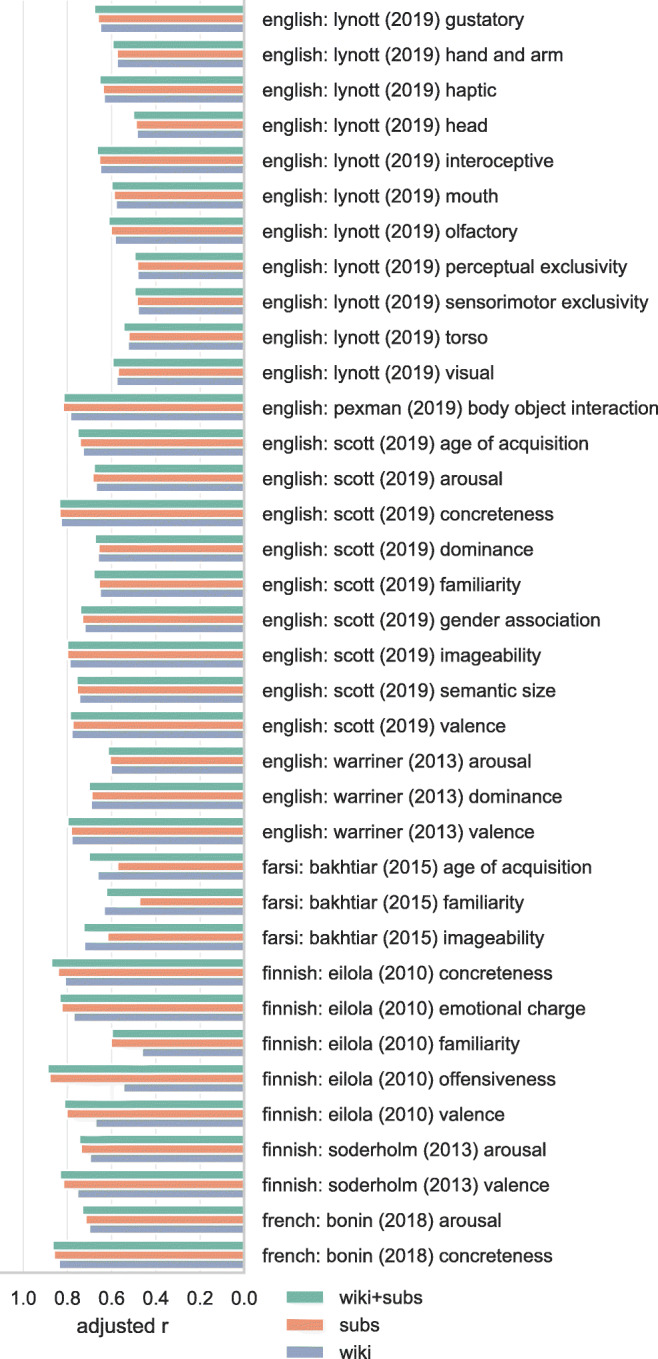
Correlations between lexical norms and our predictions for those norms based on cross-validated ridge regression using word vectors. Correlations are adjusted by penalizing for missing word vectors. 2/4

**Fig. 5:**
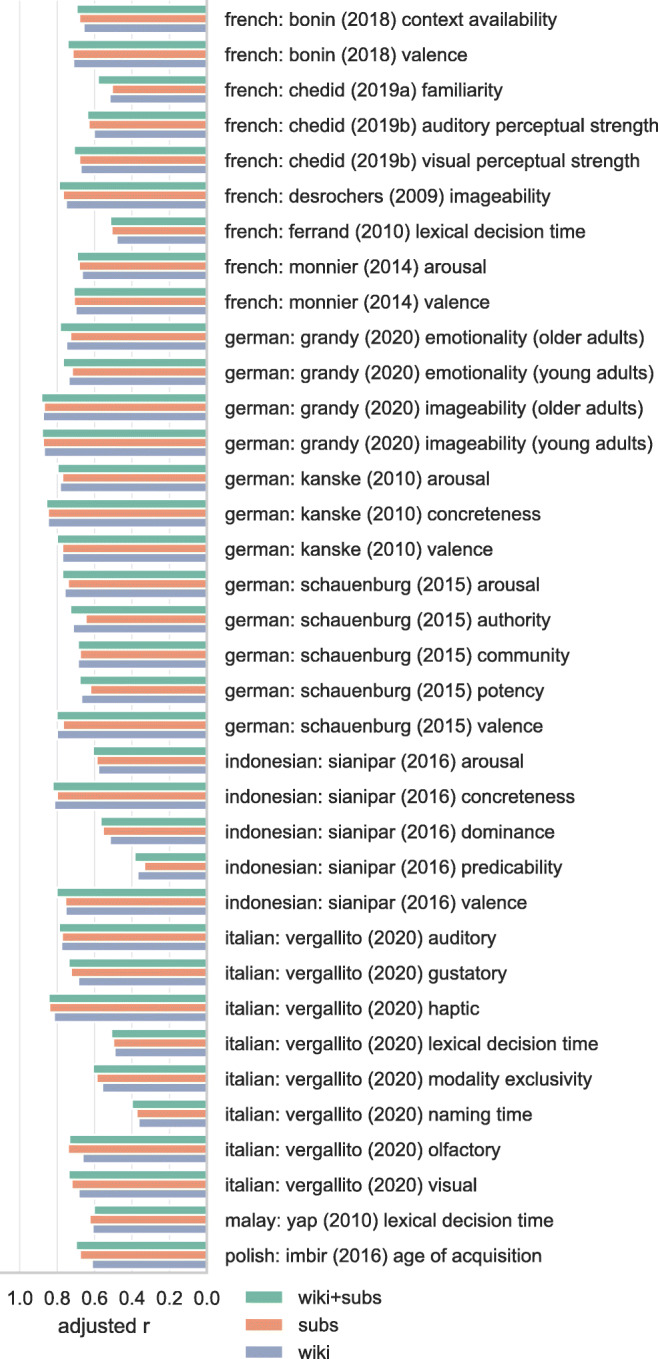
Correlations between lexical norms and our predictions for those norms based on cross-validated ridge regression using word vectors. Correlations are adjusted by penalizing for missing word vectors. 3/4

**Fig. 6:**
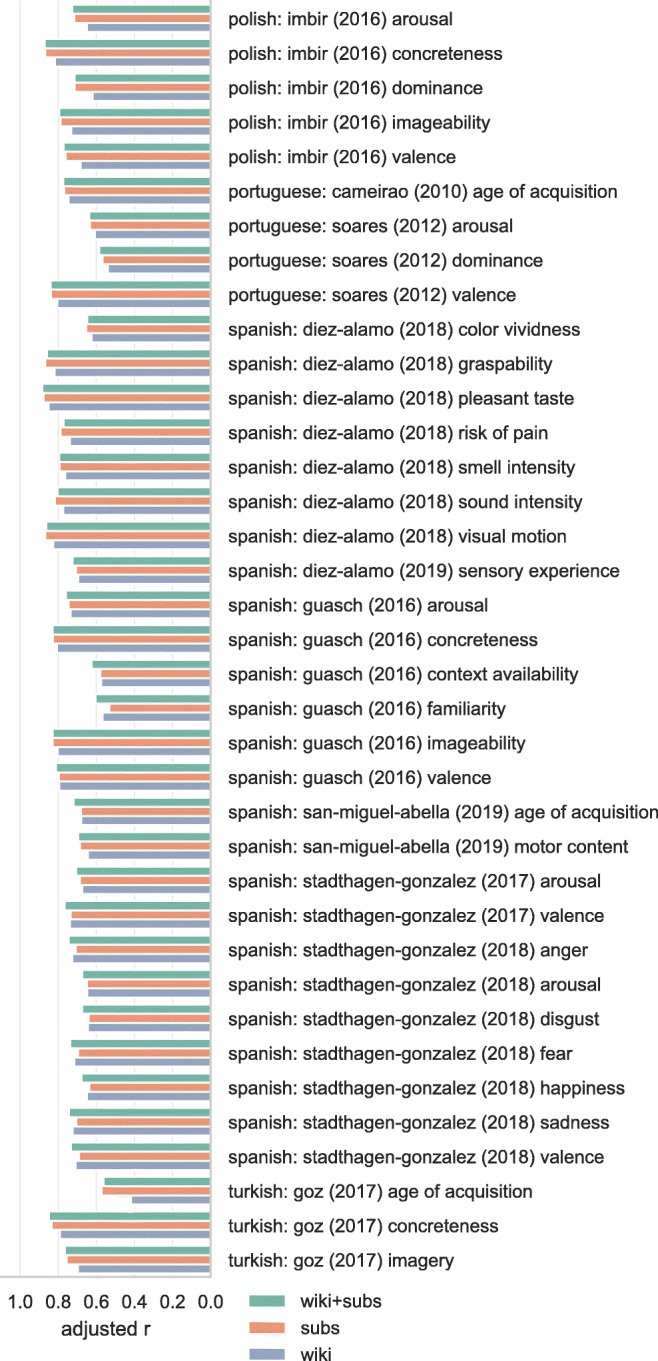
Correlations between lexical norms and our predictions for those norms based on cross-validated ridge regression using word vectors. Correlations are adjusted by penalizing for missing word vectors. 4/4

Figures 3, 4, 5, and 6 show the adjusted correlation between observed lexical norms and the norms predicted by the word embedding models. Predictive accuracy for models trained on Wikipedia and OpenSubtitles is largely similar, with a notable exception for tabooness and offensiveness, where the models trained on subtitle data perform markedly better. Offensive and taboo words are likely not represented in their usual context on Wikipedia, resulting in word vectors that do not represent the way these words are generally experienced. The subtitle vectors, while not trained on actual conversational data, capture the context in which taboo and offensive words are used much better. Models trained on a combined Wikipedia and OpenSubtitles corpus generally perform marginally better than either corpus taken separately, as predicted.

**Fig. 7:**
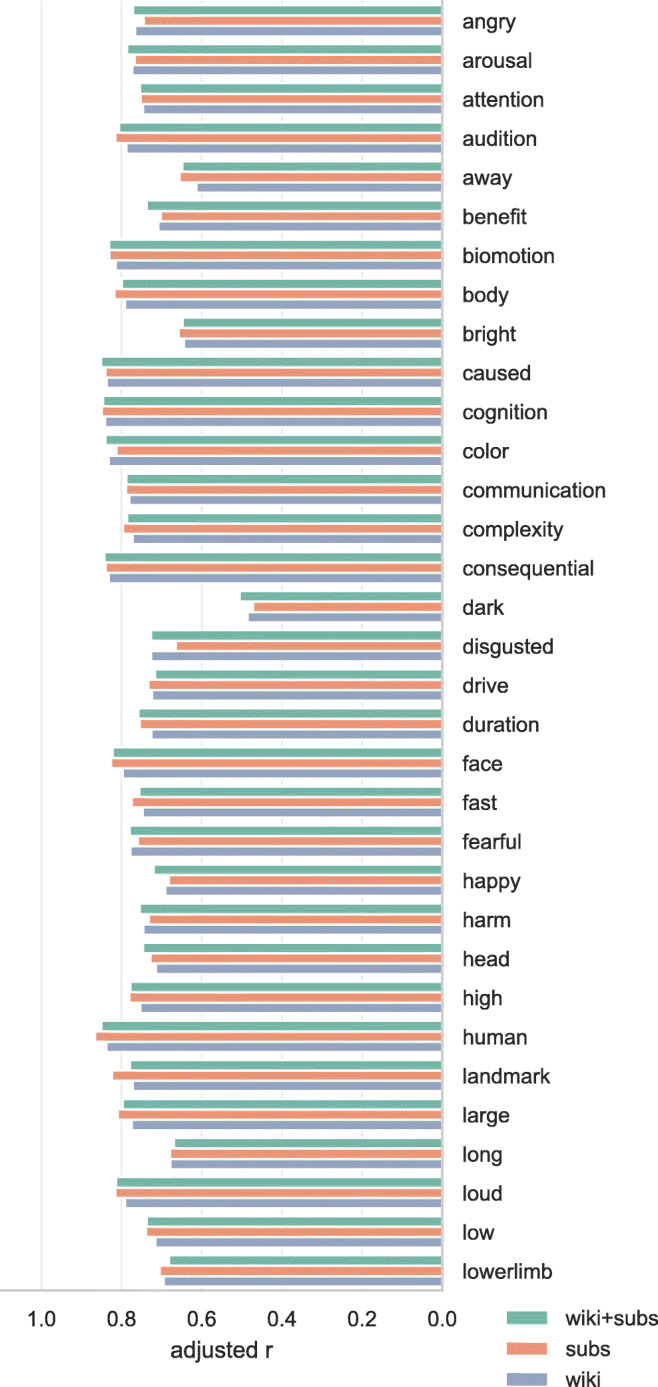
Correlations between Binder conceptual norms and our predictions for those norms based on cross-validated ridge regression using word vectors. Correlations are adjusted by penalizing for missing word vectors. 1/2

**Fig. 8:**
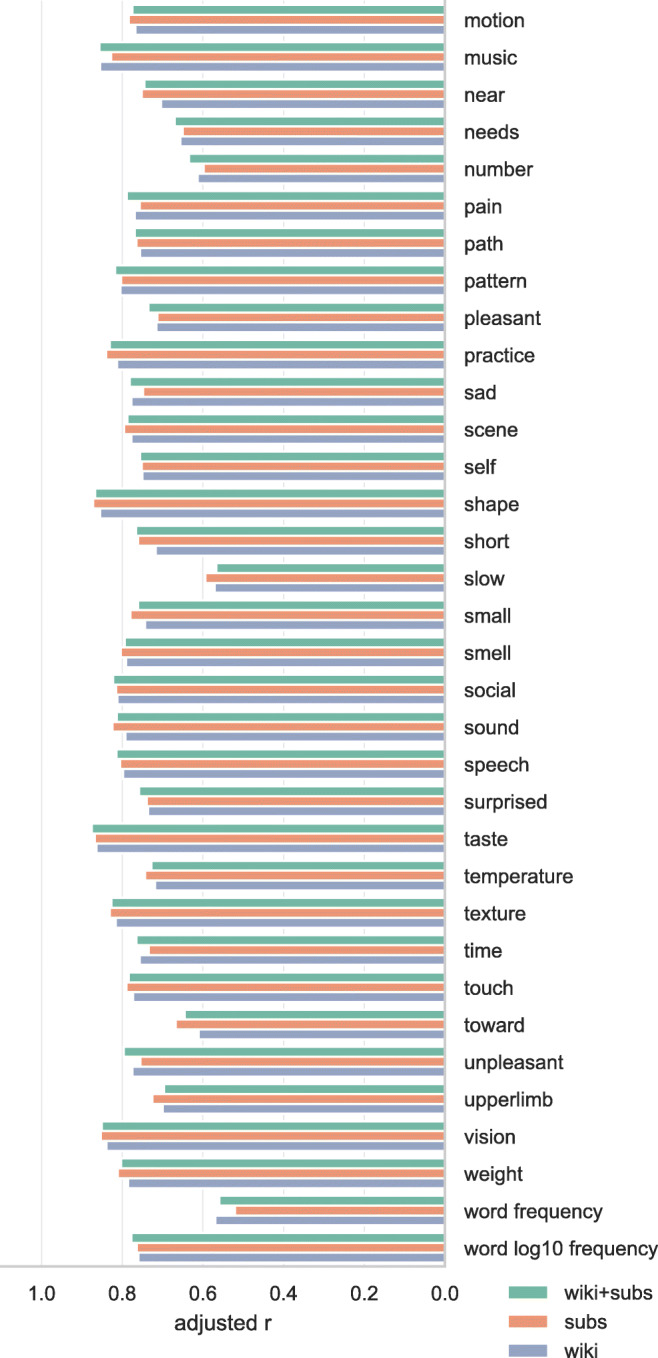
Correlations between Binder conceptual norms and our predictions for those norms based on cross-validated ridge regression using word vectors. Correlations are adjusted by penalizing for missing word vectors. 2/2

Figures 7 and 8 show the adjusted correlation between the Binder et al., (2016) conceptual norms and the norms predicted by the word embedding models. For the majority of the conceptual norms, the predictive accuracy of all three sets of word embeddings is highly similar, with little to no improvement gained from adding the OpenSubtitles and Wikipedia corpora together versus training only on either one of them. The generally high predictive value of the word embeddings for these conceptual-semantic dimensions—only for the dimensions *dark* and *slow* is the adjusted correlation for any of the sets of word embeddings lower than .6—indicates that the word embeddings are cognitively plausible, in the sense that they characterize a semantic space that is largely consistent with human ratings of semantic dimensions. The bottom two dimensions in Fig. 8 are not conceptual-semantic dimensions gathered from participant ratings, but word frequency measures. The decimal logarithm (log10) of word frequency is shown to be more predictable from the data, consistent with the generally accepted practice of log-transforming word frequencies when using them as predictors of behavior.

### Effects of pseudo-conversational versus non-conversational training data on embeddings quality

**Fig. 9:**
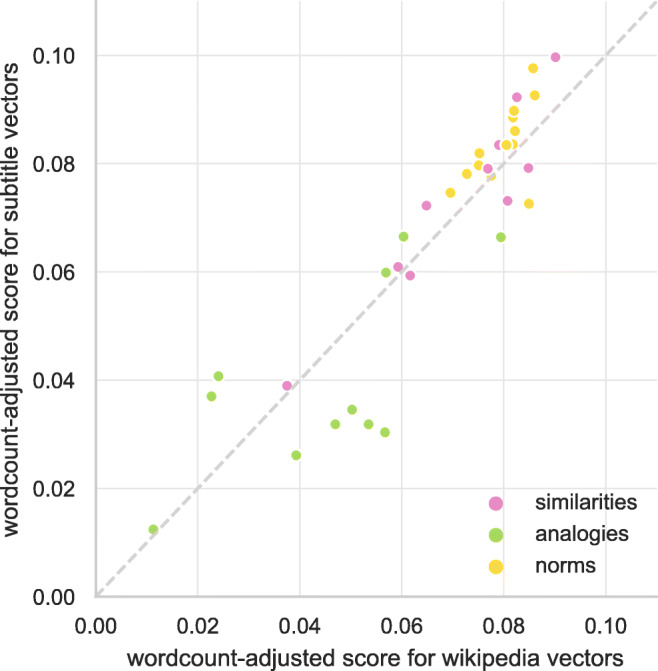
Mean evaluation scores per language and task, after correcting for training corpus size, for subtitle word embeddings versus Wikipedia word embeddings. Points above the diagonal line reflect relatively better performance for subtitle vectors than Wikipedia vectors

The Wikipedia and OpenSubtitles corpora for the various languages included in our dataset differ in size (training corpus sizes for each language are reported online at https://github.com/jvparidon/subs2vec/, where the word vectors are available for download). Because the size of the training corpus has been demonstrated to affect the quality of word embeddings (see Mandera et al., 2017, for example), it is crucial to correct for corpus size when drawing conclusions about the relative merits of subtitles versus Wikipedia as training corpora. In Fig. 9, training corpus word count-adjusted mean scores per language for each task (semantic similarities, solving analogies, and lexical norm prediction) are shown for subtitle word embeddings versus Wikipedia word embeddings. Scores were adjusted by dividing them by the log-transformed word count of their respective training corpus.

Points above the diagonal line in the figure represent relatively better performance for pseudo-conversational data, whereas points below the line represent better performance for non-conversational data. For the similarities and norms tasks, the majority of points fall above the diagonal. For the analogies, about half the points fall below the diagonal, but these points specifically represent the languages for which the semantic analogies dataset contain the aforementioned bias towards obscure geographic knowledge, whereas for all of the languages (Dutch, Greek, and Hebrew) for which we constructed a more psychologically plausible semantic dataset (the *no geo* datasets) the points fall above the diagonal. Overall, points fall fairly close to the diagonal, indicating that differences in performance between the subtitle and Wikipedia embeddings are relatively minor.

To test the effect of the different training corpora on embedding quality statistically, we conducted a Bayesian multilevel Beta regression, with training corpus size, training corpus type, evaluation task, and the interaction of training corpus type and evaluation task as fixed effects and language and specific evaluation dataset as random intercepts. Priors on all reported coefficients were set to $\mathcal {N}(0, 1)$, a mild shrinkage prior. We implemented this model in PyMC3, and sampled from it using the No-U-Turn Sampler (Salvatier et al., 2016; Hoffman and Gelman, 2014). We ran 4 chains for 2500 warmup samples each, followed by 2500 true posterior samples each (for a total of 10,000 posterior samples). Sampler diagnostics were all within acceptable limits (no divergences, $\hat {r}$ below 1.01 and at least 1000 effective samples for all parameters. Further details on the inferential model, such as a directed acyclic graph of the model and trace summaries, are reported in Appendix app:modelA.

**Fig. 10:**
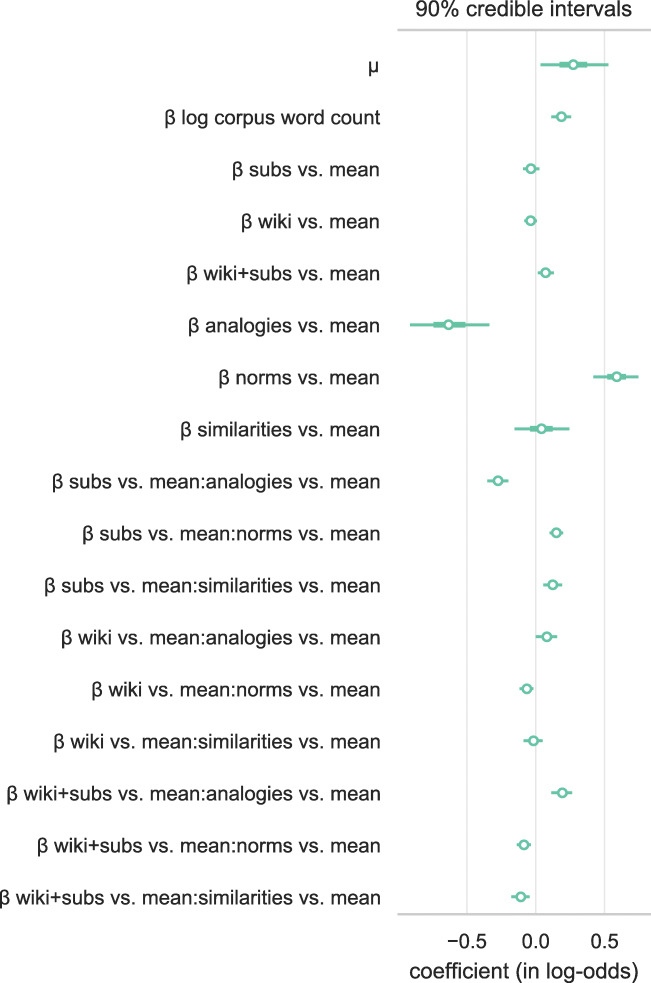
Posterior estimates from Beta regression model of OpenSubtitles and Wikipedia embeddings performance on our evaluation tasks. Beta regression uses a logit link function, therefore coefficients can be interpreted similarly to coefficients in other logit-link regressions (e.g., logistic regression). Model uses effects coding for the contrast; for example, *subs vs. mean* indicates the performance of subtitle-based embeddings relative to the mean performance of all three sets of embeddings

This regression analysis demonstrates that after correcting for size of training corpus, subtitle embeddings are virtually indistinguishable from Wikipedia embeddings (or combined subtitle and Wikipedia embeddings) in terms of overall embedding quality (see Fig. 10 for coefficient estimates). As is to be expected, the aforementioned advantage of a training corpus containing Wikipedia for solving geographic analogies is visible in the interaction estimates as well.

## Discussion

Our aim in this study was to make available a collection of word embeddings trained on pseudo-conversational language in as many languages as possible using the same algorithm. We introduced vector embeddings in 55 languages, trained using the fastText implementation of the skipgram algorithm on the OpenSubtitles dataset. We selected the fastText algorithm because (1) it represents the state of the art in word embedding algorithms at the time of writing; and (2) there is an efficient, easy-to-use, and open-source implementation of the algorithm. In order to evaluate the performance of these vectors, we also trained vector embeddings on Wikipedia, and on a combination of Wikipedia and subtitles, using the same algorithm. We evaluated all of these embeddings on standard benchmark tasks. In response to the limitations of these standard evaluation tasks (Faruqui et al., 2016), we curated a dataset of multilingual lexical norms and evaluated all vector embeddings on their ability to accurately predict these ratings. We have made all of these materials, including utilities to easily obtain preprocessed versions of the original training datasets (and derived word, bigram, and trigram frequencies), available online at https://github.com/jvparidon/subs2vec/. These materials include the full binary representations of the embeddings we trained in addition to plain-text vector representations. The binaries can be used to compute embeddings for out-of-sample vocabulary, allowing other researchers to explore the embeddings beyond the analyses reported here.

### Performance and evaluation

Contrary to our expectations, conversational embeddings did not generally outperform alternative embeddings at predicting human lexical judgments (this contrasts with previously published predictions as well, see e.g. Mandera et al., (2017), p. 75). Our evaluation of embeddings trained on pseudo-conversational speech transcriptions (OpenSubtitles) showed that they exhibit performance rates similar to those exhibited by embeddings trained on a highly structured, knowledge-rich dataset (Wikipedia). This attests to the structured lexical relationships implicit in conversational language. However, we also suspect that more nuanced evaluation methods would reveal more substantive differences between the representations induced from these corpora. Vectors trained on pseudo-conversational text consistently outperformed vectors trained on encyclopedic text in predicting lexical judgments relating to offensiveness or tabooness, but underperformed the alternative in solving knowledge-based semantic analogies in the geographic domain (e.g. relationships between countries and capital cities). Neither of these evaluation tasks were explicitly chosen by us because they were intended to be diagnostic of one particular kind of linguistic experience, but it is notable that tabooness and offensiveness of common insults for instance are common knowledge, whereas the relationship between small countries and their respective currencies is not something the average person would know, and therefore a poor test of cognitive plausibility. The development of evaluation tasks that are independently predicted to be solvable after exposure to conversational language merits further study.

Unfortunately, we were not able to compile evaluation metrics for every one of the 55 languages in which we provide embeddings. We did locate suitable evaluation datasets for 19 languages (and in many of these cases we provide multiple different evaluation datasets per language). That leaves embeddings in 36 languages for which we could not locate suitable evaluation datasets. This does not preclude the use of these embeddings, but we recommend researchers use them with appropriate caution, specifically by taking into account the size of the corpus that embeddings were trained on (see Appendix app:corporaB).

Overall, we found that embeddings trained on a combination of Wikipedia and OpenSubtitles generally outperformed embeddings trained on either of those corpora individually, even after accounting for corpus size. We hypothesize this is because the subtitle and Wikipedia embeddings represent separate, but overlapping semantic spaces, which can be jointly characterized by embeddings trained on a combined corpus. Taking into account the effect of corpus size, we recommend researchers use the embeddings trained on the largest and most diverse corpus available (subtitles plus Wikipedia, in the present study), unless they have hypotheses specific to embeddings trained on a conversational corpus.

### Extending language coverage through complementary multilingual corpora

Our primary aim for the present study was to produce embeddings in multiple languages trained on a dataset that is more naturalistic than the widely available alternatives in multiple languages (embeddings trained on Wikipedia and other text scraped from the internet). However, it also contributes to the availability and quality of word vectors for underrepresented and less studied languages. Specifically, in some of these languages, the corresponding corpus of Wikipedia articles is small or of low quality, while the OpenSubtitles corpus is substantially larger (e.g., Bulgarian, 4x larger; Bosnian, 7x larger; Greek, 5x larger; Croatian, 6x larger; Romanian, 7x larger; Serbian, 5x larger; Turkish, 4x larger). As a result, our study helps to increase the number of languages for which high quality embeddings are available, regardless of whether the pseudo-conversational nature of the training corpus is germane to the specific purpose for which the embeddings may be used.

### Translation vs. original language

An important caveat in using the OpenSubtitles corpus in the present context is that many of the subtitles are translations, meaning the subtitles are not straight transcriptions, but a translation from speech in the original language a movie or television series was released in to text in another language. Moreover, while it is highly likely that translators try to produce subtitles that are correct and coherent in the target language, we have no reliable way of ascertaining the proficiency of the (often anonymous) translator in either source or language. In the present context it was not feasible to examine which parts of the subtitle corpus are translations and which represent straight transcriptions of audio in the original language and therefore we could not test whether training on translated subtitles has an adverse effect on word embedding quality. This issue is not unsolvable in principle, because the original language of the movies and television series for which each set of subtitles was written can be established using secondary, publicly available datasets. Future work investigating distributional differences between transcribed and translated dialogue seems warranted.

A related ambiguity is whether subtitles should be viewed as representing experience of written or spoken language. On the one hand, subtitles are read by many people. However, as transcriptions of speech, subtitles convey a more direct representation of spoken language experience than is conveyed by other written corpora such as Wikipedia. This second interpretation was an important part of our motivation, but the interpretation of subtitles as written language is also important.

### Advances in fastText algorithms

The most recent implementation of the fastText algorithm includes CBOW with position-dependent weighting of the context vectors, which seems to represent another step forward in terms of the validity of the word embeddings it generates (Mikolov et al., 2018). As of the time of writing, this implementation has not been released to the public (although a rudimentary description of the algorithm has been published, alongside a number of word vector datasets in various languages created using the new version of the algorithm). Because all the code used in the present study is publicly available, if and when an implementation of the new algorithm is released to the public, the present study and dataset can easily be reproduced using this improved method for computing word vectors.

Algorithmic developments in the field of distributional semantics move quickly. Nonetheless, in this paper we have produced (for a large set of languages, using state of the art methods) word embeddings trained on a large corpus of language that reflects real-world linguistic experience. In addition to insights about language and cognition that can be gleaned from these embeddings directly, they are a valuable resource for improving statistical models of other psychological and linguistic phenomena.

## Open practices statement

All of the datasets and code presented in this paper, as well as the datasets and code necessary to reproduce the analyses, are freely available online at https://github.com/jvparidon/subs2vec/.

The *subs2vec* Python package also provides tools can be used to compute semantic dissimilarities, solve analogies, and predict lexical norms for novel datasets.

## Figures and Tables

**Fig. 11:**
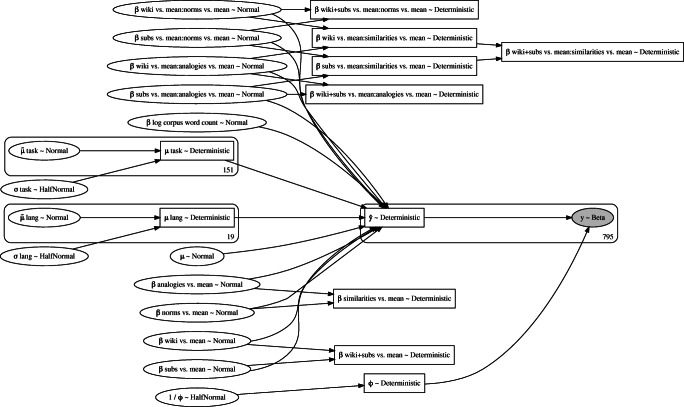
Directed acyclic graph of inferential model, node labels include shape of prior distribution. Random intercepts were estimated by language, but also by evaluation task where appropriate (e.g., the MC30 similarities were used in Arabic, Dutch, English, Romanian, and Spanish). The likelihood uses the Beta(*μ*,*ϕ*) parametrization of the Beta distribution. Coefficients labeled ”Deterministic” follow trivially from the other coefficient estimates and were computed during model estimation

**Fig. 12:**
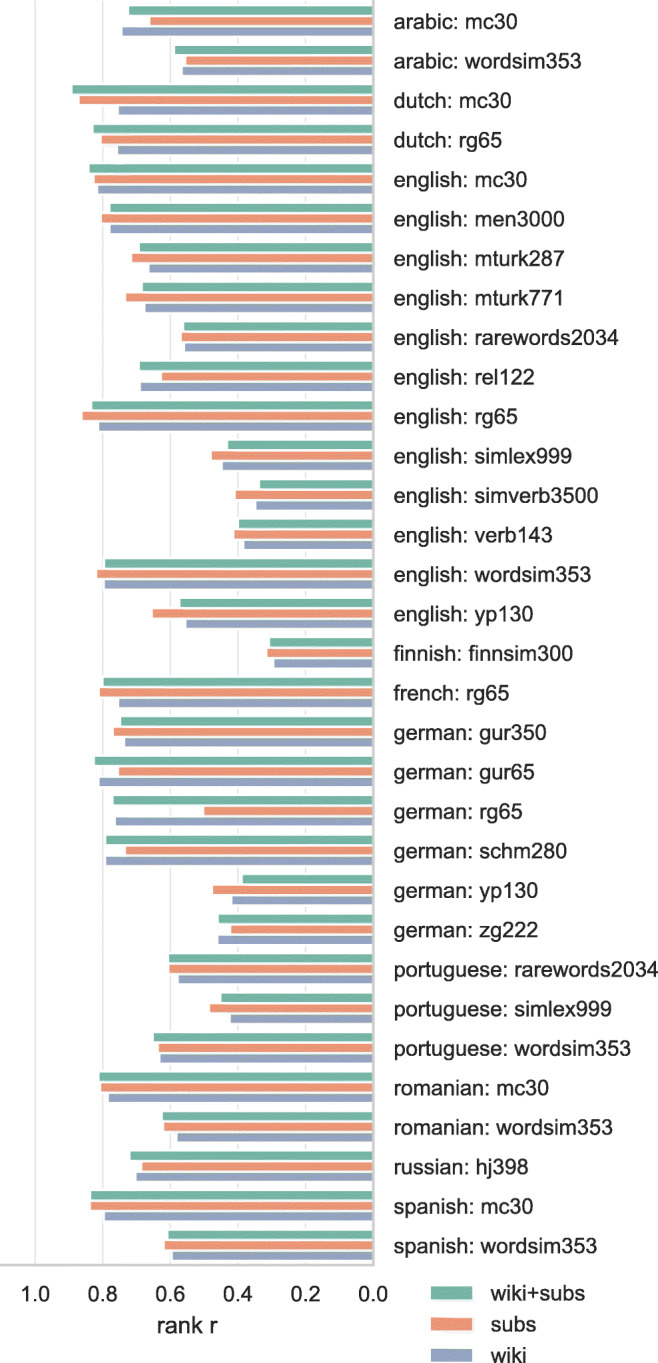
Unpenalized rank correlations between human ratings of semantic similarity and word vector cosine similarity

**Fig. 13:**
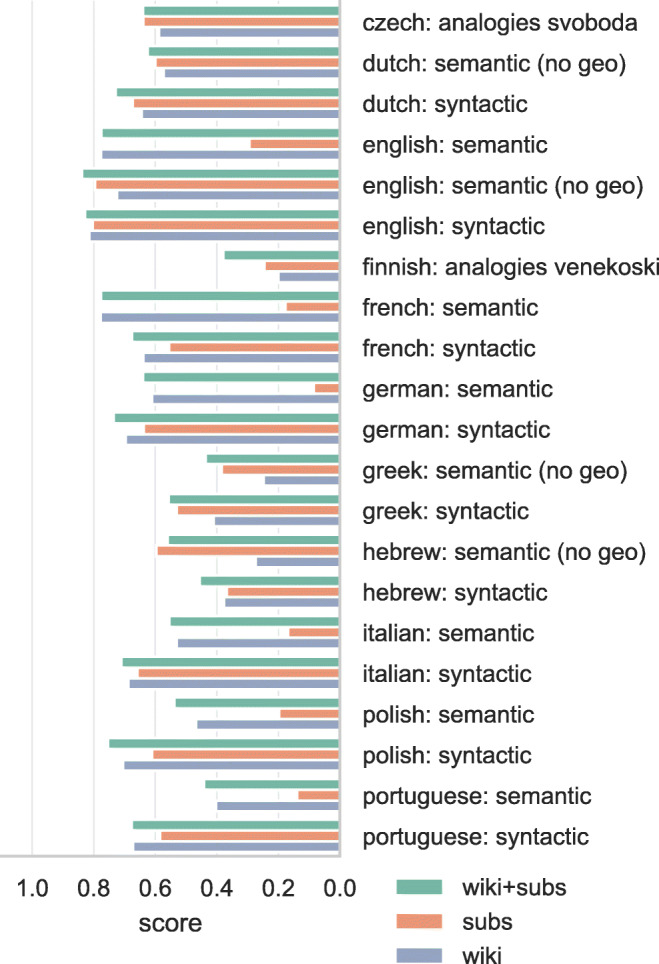
Unpenalized proportion of correctly solved analogies in the semantic and syntactic domain using word vectors. Semantic datasets contained 93% geographic analogies, *no geo* datasets are those same datasets, excluding the geographic analogies

**Fig. 14:**
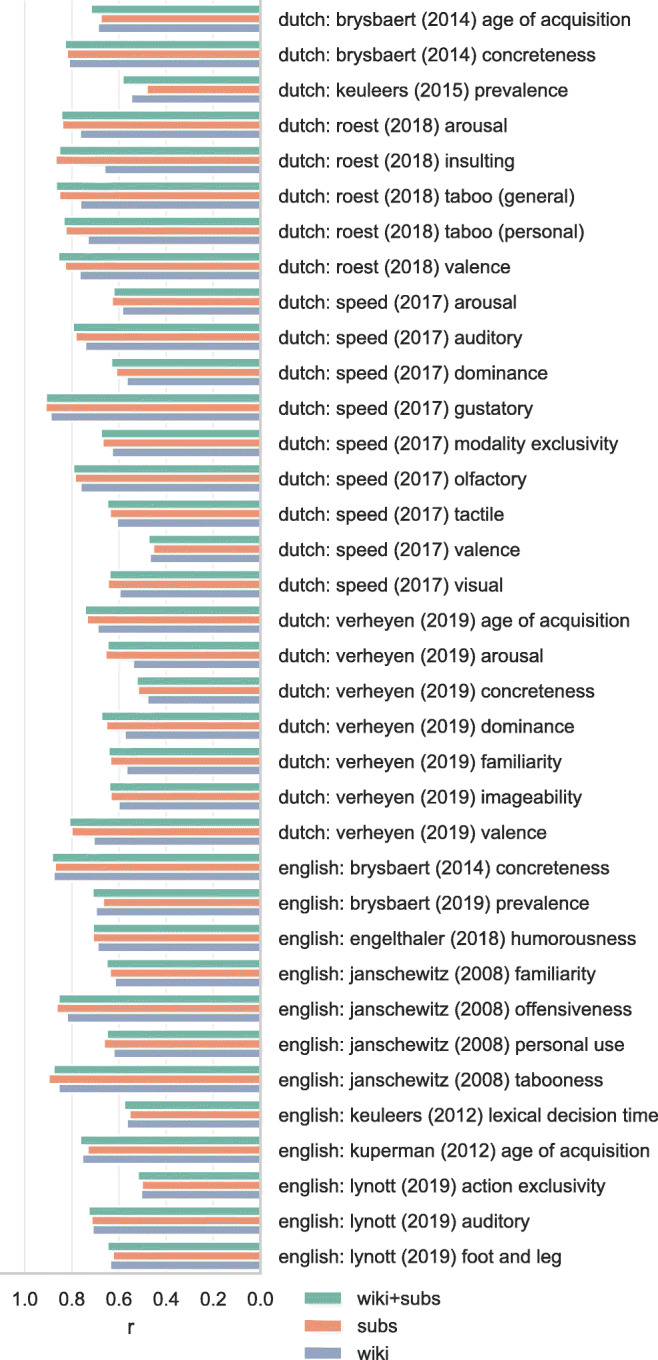
Unpenalized correlations between lexical norms and our predictions for those norms based on cross-validated ridge regression using word vectors. 1/4

**Fig. 15:**
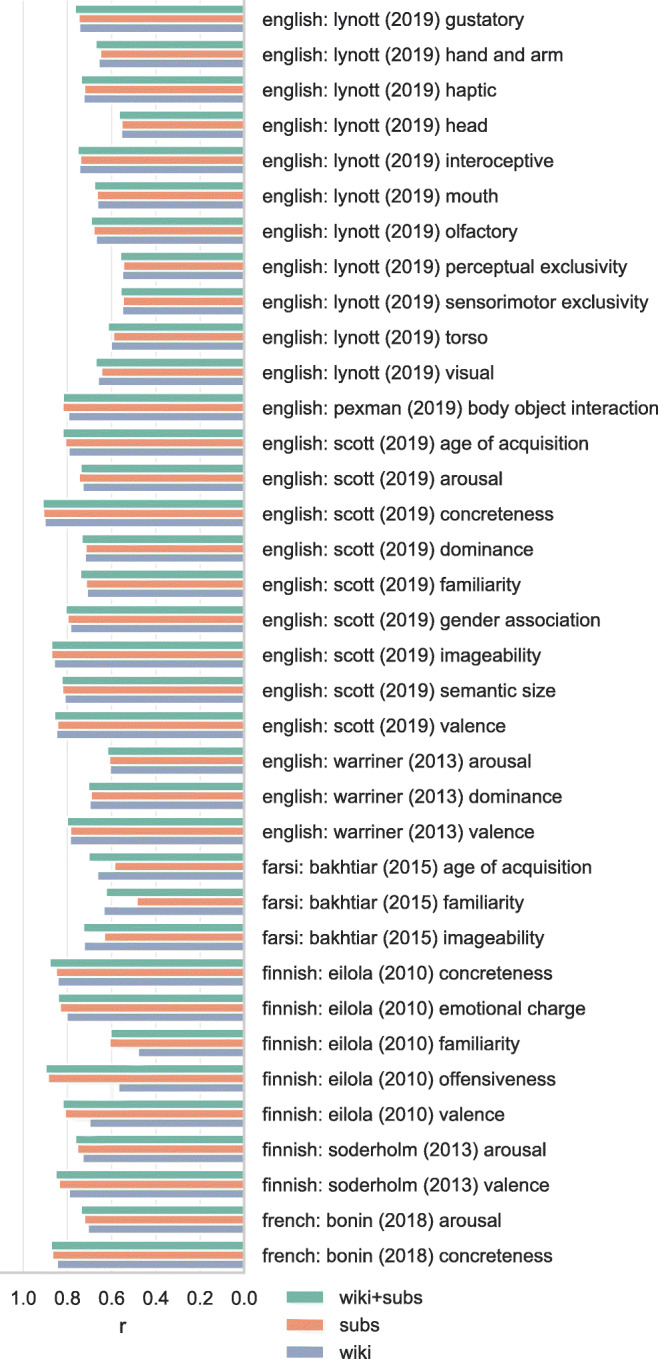
Unpenalized correlations between lexical norms and our predictions for those norms based on cross-validated ridge regression using word vectors. 2/4

**Fig. 16:**
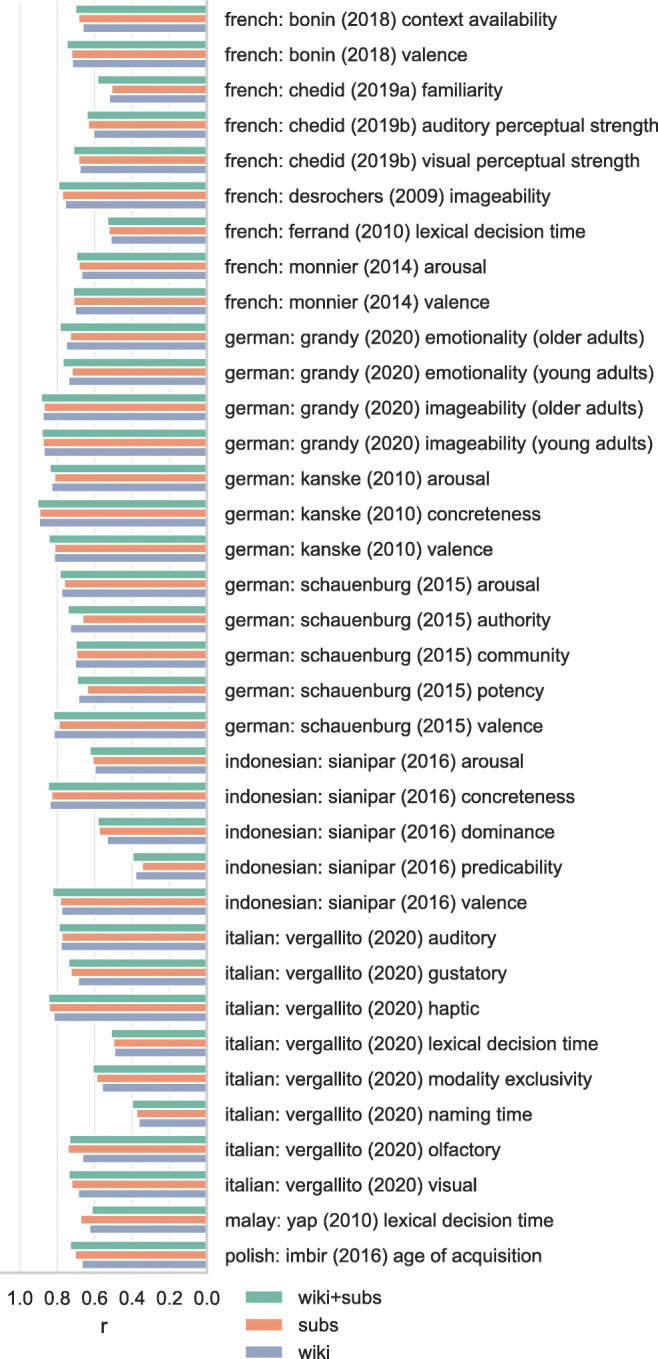
Unpenalized correlations between lexical norms and our predictions for those norms based on cross-validated ridge regression using word vectors. 3/4

**Fig. 17:**
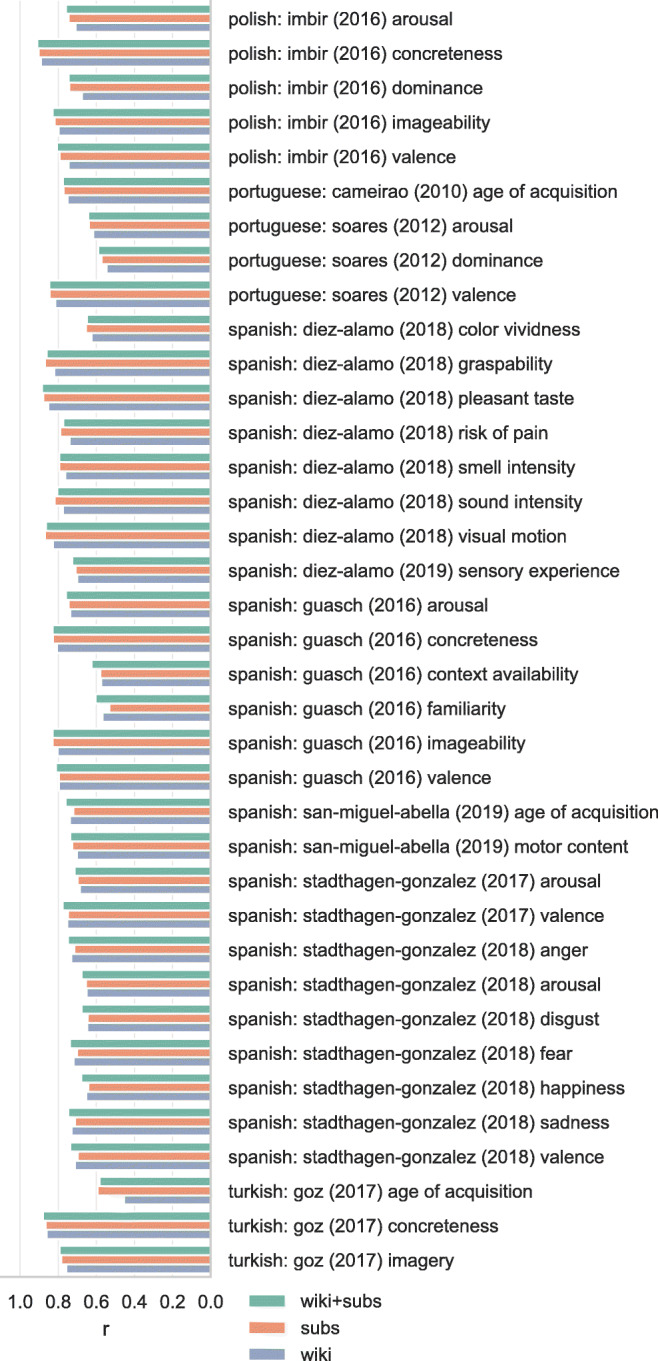
Unpenalized correlations between lexical norms and our predictions for those norms based on cross-validated ridge regression using word vectors. 4/4

**Fig. 18:**
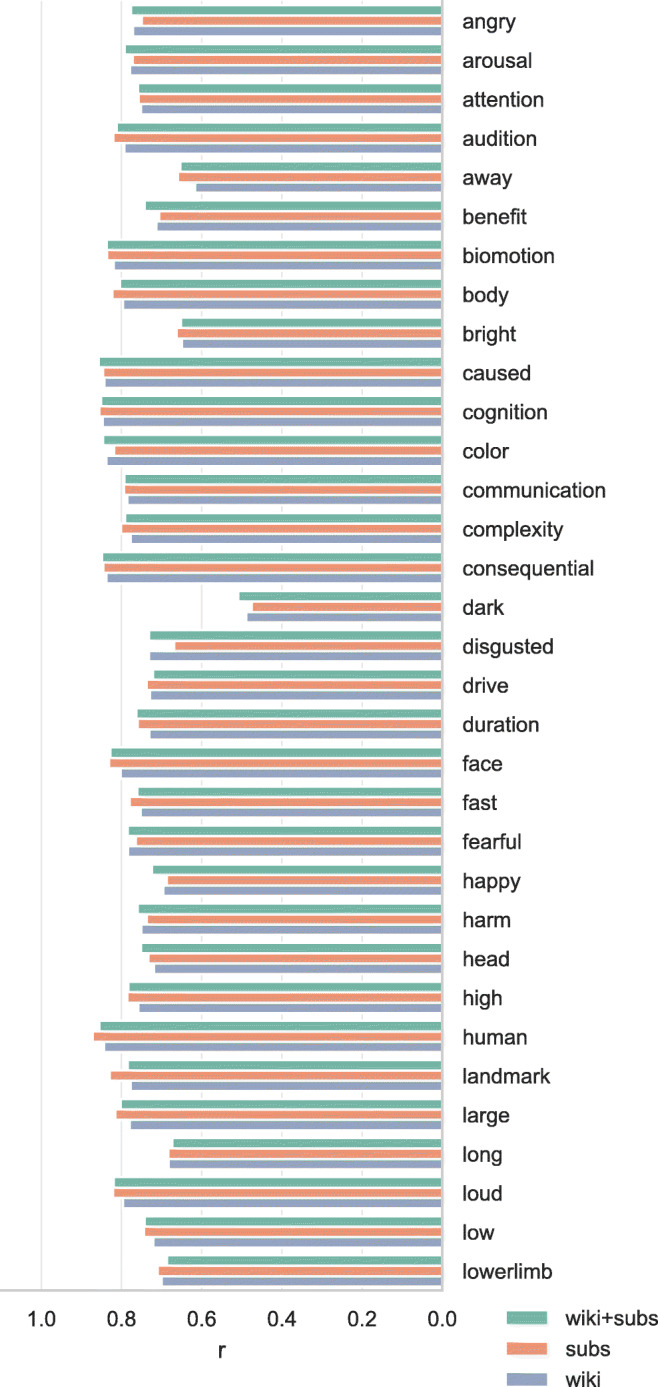
Unpenalized correlations between Binder conceptual norms and our predictions for those norms based on cross-validated ridge regression using word vectors. 1/2

**Fig. 19:**
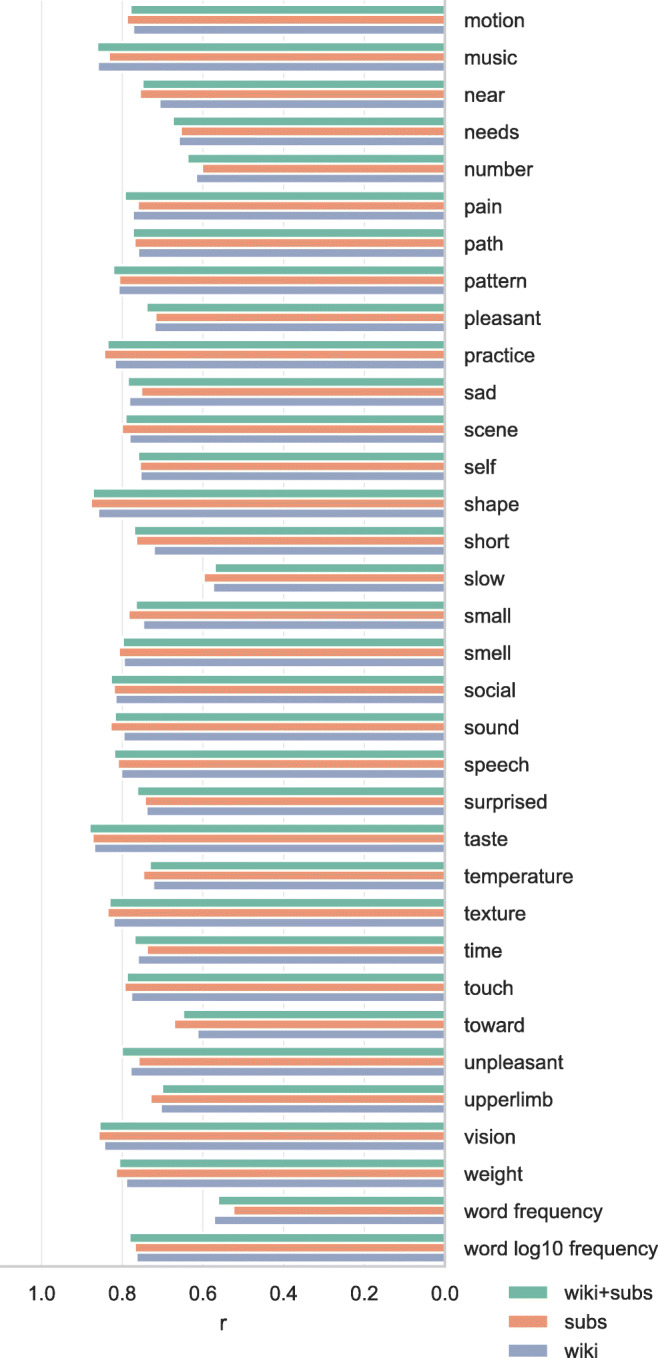
Unpenalized correlations between Binder conceptual norms and our predictions for those norms based on cross-validated ridge regression using word vectors. 2/2

## Appendix A: Inferential model details

**Table 4:** Summary of posterior traces for inferential model

	mean	sd	90% CI lower	90% CI upper	MCSE mean	MCSE sd	*n*_eff_ mean	*n*_eff_ sd	*n*_eff_ bulk	*n*_eff_ tail	$\hat {r}$
*μ*	0.28	0.15	0.04	0.51	0.00	0.00	1382.0	1382.0	1384.0	2724.0	1.00
*β* log corpus word count	0.19	0.04	0.12	0.26	0.00	0.00	4159.0	4159.0	4161.0	5831.0	1.00
*β* wiki vs. mean	− 0.04	0.03	− 0.08	0.01	0.00	0.00	4716.0	4716.0	4720.0	6273.0	1.00
*β* subs vs. mean	− 0.04	0.04	− 0.09	0.02	0.00	0.00	4237.0	4237.0	4238.0	5759.0	1.00
*β* norms vs. mean	0.59	0.10	0.43	0.76	0.00	0.00	1680.0	1672.0	1682.0	3048.0	1.00
*β* analogies vs. mean	− 0.62	0.17	− 0.90	− 0.34	0.00	0.00	2200.0	2200.0	2202.0	3547.0	1.00
*β* wiki vs. mean:norms vs. mean	− 0.06	0.03	− 0.12	− 0.02	0.00	0.00	4425.0	4425.0	4423.0	5445.0	1.00
*β* wiki vs. mean:analogies vs. mean	0.08	0.05	0.01	0.16	0.00	0.00	5729.0	5729.0	5730.0	6317.0	1.00
*β* subs vs. mean:norms vs. mean	0.15	0.03	0.10	0.20	0.00	0.00	4977.0	4977.0	4980.0	6464.0	1.00
*β* subs vs. mean:analogies vs. mean	− 0.27	0.05	− 0.35	− 0.20	0.00	0.00	6110.0	6104.0	6108.0	6592.0	1.00
*β* wiki+subs vs. mean	0.07	0.04	0.01	0.13	0.00	0.00	6077.0	5947.0	6077.0	6521.0	1.00
*β* similarities vs. mean	0.04	0.12	− 0.16	0.24	0.00	0.00	1986.0	1986.0	1984.0	3972.0	1.00
*β* wiki+subs vs. mean:norms vs. mean	− 0.08	0.03	− 0.14	− 0.04	0.00	0.00	12024.0	11067.0	12020.0	7926.0	1.00
*β* wiki+subs vs. mean:analogies vs. mean	0.19	0.05	0.11	0.26	0.00	0.00	14384.0	13239.0	14376.0	8151.0	1.00
*β* subs vs. mean:similarities vs. mean	0.12	0.04	0.05	0.19	0.00	0.00	13000.0	11975.0	12988.0	8261.0	1.00
*β* wiki vs. mean:similarities vs. mean	− 0.02	0.04	− 0.09	0.05	0.00	0.00	13382.0	6400.0	13411.0	8037.0	1.00
*β* wiki+subs vs. mean:similarities vs. mean	− 0.11	0.04	− 0.18	− 0.04	0.00	0.00	18032.0	14781.0	18151.0	7692.0	1.00
*σ* task	0.52	0.04	0.46	0.58	0.00	0.00	1907.0	1907.0	1903.0	3917.0	1.00
*σ* lang	0.45	0.10	0.28	0.61	0.00	0.00	2503.0	2503.0	2484.0	4736.0	1.00
*ϕ*	34.65	1.96	31.44	37.87	0.02	0.02	6631.0	6601.0	6688.0	6692.0	1.00

90% CI upper and lower refer to upper and lower bounds of the credible interval. MCSE refers to Markov chain standard error, *n*_eff_ is the estimated effective sample size

## Appendix B: Training corpus details

**Table 5:** Descriptive statistics for training corpora

Language	Corpus	Word count	Mean words per line
Afrikaans	OpenSubtitles	324K	6.61
	Wikipedia	17M	17.01
	Wikipedia + OpenSubtitles	17M	16.53
Albanian	OpenSubtitles	12M	6.65
	Wikipedia	18M	16.90
	Wikipedia + OpenSubtitles	30M	10.47
Arabic	OpenSubtitles	188M	5.64
	Wikipedia	120M	18.32
	Wikipedia + OpenSubtitles	308M	7.72
Armenian	OpenSubtitles	24K	6.06
	Wikipedia	38M	21.66
	Wikipedia + OpenSubtitles	39M	21.62
Basque	OpenSubtitles	3M	4.97
	Wikipedia	20M	11.39
	Wikipedia + OpenSubtitles	24M	9.60
Bengali	OpenSubtitles	2M	5.39
	Wikipedia	19M	27.64
	Wikipedia + OpenSubtitles	21M	19.16
Bosnian	OpenSubtitles	92M	6.34
	Wikipedia	13M	13.15
	Wikipedia + OpenSubtitles	105M	6.78
Breton	OpenSubtitles	111K	5.97
	Wikipedia	8M	15.72
	Wikipedia + OpenSubtitles	8M	15.36
Bulgarian	OpenSubtitles	247M	6.87
	Wikipedia	53M	15.82
	Wikipedia + OpenSubtitles	300M	7.64
Catalan	OpenSubtitles	3M	6.95
	Wikipedia	176M	20.75
	Wikipedia + OpenSubtitles	179M	20.06
Croatian	OpenSubtitles	242M	6.44
	Wikipedia	43M	12.25
	Wikipedia + OpenSubtitles	285M	6.94
Czech	OpenSubtitles	249M	6.43
	Wikipedia	100M	13.44
	Wikipedia + OpenSubtitles	349M	7.57
Danish	OpenSubtitles	87M	6.96
	Wikipedia	56M	14.72
	Wikipedia + OpenSubtitles	143M	8.77
Dutch	OpenSubtitles	265M	7.39
	Wikipedia	249M	14.40
	Wikipedia + OpenSubtitles	514M	9.67
English	OpenSubtitles	751M	8.22
	Wikipedia	2B	17.57
	Wikipedia + OpenSubtitles	3B	13.90
Esperanto	OpenSubtitles	382K	5.44
	Wikipedia	38M	14.64
	Wikipedia + OpenSubtitles	38M	14.39
Estonian	OpenSubtitles	60M	5.99
	Wikipedia	29M	10.38
	Wikipedia + OpenSubtitles	90M	6.94
Farsi	OpenSubtitles	45M	6.39
	Wikipedia	87M	17.36
	Wikipedia + OpenSubtitles	132M	10.92
Finnish	OpenSubtitles	117M	5.10
	Wikipedia	74M	10.80
	Wikipedia + OpenSubtitles	191M	6.40
French	OpenSubtitles	336M	8.31
	Wikipedia	724M	19.54
	Wikipedia + OpenSubtitles	1B	13.69
Galician	OpenSubtitles	2M	6.58
	Wikipedia	40M	18.56
	Wikipedia + OpenSubtitles	42M	17.30
Georgian	OpenSubtitles	1M	5.21
	Wikipedia	15M	11.04
	Wikipedia + OpenSubtitles	16M	10.26
German	OpenSubtitles	139M	7.01
	Wikipedia	976M	14.06
	Wikipedia + OpenSubtitles	1B	12.49
Greek	OpenSubtitles	271M	6.90
	Wikipedia	58M	18.26
	Wikipedia + OpenSubtitles	329M	7.76
Hebrew	OpenSubtitles	170M	6.22
	Wikipedia	133M	13.92
	Wikipedia + OpenSubtitles	303M	8.22
Hindi	OpenSubtitles	660K	6.77
	Wikipedia	31M	33.89
	Wikipedia + OpenSubtitles	32M	31.28
Hungarian	OpenSubtitles	228M	6.04
	Wikipedia	121M	12.37
	Wikipedia + OpenSubtitles	349M	7.34
Icelandic	OpenSubtitles	7M	6.08
	Wikipedia	7M	13.17
	Wikipedia + OpenSubtitles	15M	8.26
Indonesian	OpenSubtitles	65M	6.18
	Wikipedia	69M	14.09
	Wikipedia + OpenSubtitles	134M	8.70
Italian	OpenSubtitles	278M	7.43
	Wikipedia	476M	18.87
	Wikipedia + OpenSubtitles	754M	12.05
Kazakh	OpenSubtitles	13K	3.90
	Wikipedia	18M	10.39
	Wikipedia + OpenSubtitles	18M	10.38
Korean	OpenSubtitles	7M	4.30
	Wikipedia	63M	11.97
	Wikipedia + OpenSubtitles	70M	10.19
Estonian	OpenSubtitles	60M	5.99
	Wikipedia	29M	10.38
	Wikipedia + OpenSubtitles	90M	6.94
Farsi	OpenSubtitles	45M	6.39
	Wikipedia	87M	17.36
	Wikipedia + OpenSubtitles	132M	10.92
Finnish	OpenSubtitles	117M	5.10
	Wikipedia	74M	10.80
	Wikipedia + OpenSubtitles	191M	6.40
French	OpenSubtitles	336M	8.31
	Wikipedia	724M	19.54
	Wikipedia + OpenSubtitles	1B	13.69
Galician	OpenSubtitles	2M	6.58
	Wikipedia	40M	18.56
	Wikipedia + OpenSubtitles	42M	17.30
Latvian	OpenSubtitles	2M	5.10
	Wikipedia	14M	10.91
	Wikipedia + OpenSubtitles	16M	9.46
Lithuanian	OpenSubtitles	6M	4.89
	Wikipedia	23M	11.10
	Wikipedia + OpenSubtitles	29M	8.74
Macedonian	OpenSubtitles	20M	6.33
	Wikipedia	27M	16.82
	Wikipedia + OpenSubtitles	47M	9.82
Malay	OpenSubtitles	12M	5.88
	Wikipedia	29M	14.50
	Wikipedia + OpenSubtitles	41M	10.11
Malayalam	OpenSubtitles	2M	4.08
	Wikipedia	10M	9.18
	Wikipedia + OpenSubtitles	12M	7.92
Norwegian	OpenSubtitles	46M	6.69
	Wikipedia	91M	14.53
	Wikipedia + OpenSubtitles	136M	10.44
Polish	OpenSubtitles	250M	6.15
	Wikipedia	232M	12.63
	Wikipedia + OpenSubtitles	483M	8.17
Portuguese	OpenSubtitles	258M	7.40
	Wikipedia	238M	18.60
	Wikipedia + OpenSubtitles	496M	10.41
Romanian	OpenSubtitles	435M	7.70
	Wikipedia	65M	16.16
	Wikipedia + OpenSubtitles	500M	8.27
Russian	OpenSubtitles	152M	6.43
	Wikipedia	391M	13.96
	Wikipedia + OpenSubtitles	543M	10.51
Serbian	OpenSubtitles	344M	6.57
	Wikipedia	70M	12.97
	Wikipedia + OpenSubtitles	413M	7.16
Sinhala	OpenSubtitles	3M	5.34
	Wikipedia	6M	14.52
	Wikipedia + OpenSubtitles	9M	8.89
Slovak	OpenSubtitles	47M	6.23
	Wikipedia	29M	12.85
	Wikipedia + OpenSubtitles	76M	7.73
Slovenian	OpenSubtitles	107M	6.15
	Wikipedia	32M	13.45
	Wikipedia + OpenSubtitles	138M	7.02
Spanish	OpenSubtitles	514M	7.46
	Wikipedia	586M	20.36
	Wikipedia + OpenSubtitles	1B	11.25
Swedish	OpenSubtitles	101M	6.87
	Wikipedia	143M	11.93
	Wikipedia + OpenSubtitles	245M	9.15
Tagalog	OpenSubtitles	88K	6.02
	Wikipedia	7M	17.16
	Wikipedia + OpenSubtitles	7M	16.74
Tamil	OpenSubtitles	123K	4.36
	Wikipedia	17M	10.09
	Wikipedia + OpenSubtitles	17M	10.00
Telugu	OpenSubtitles	103K	4.50
	Wikipedia	15M	10.34
	Wikipedia + OpenSubtitles	15M	10.25
Turkish	OpenSubtitles	240M	5.56
	Wikipedia	55M	12.52
	Wikipedia + OpenSubtitles	295M	6.20
Ukrainian	OpenSubtitles	5M	5.51
	Wikipedia	163M	13.34
	Wikipedia + OpenSubtitles	168M	12.80
Urdu	OpenSubtitles	196K	7.02
	Wikipedia	16M	28.88
	Wikipedia + OpenSubtitles	16M	27.83
Vietnamese	OpenSubtitles	27M	8.23
	Wikipedia	115M	20.51
	Wikipedia + OpenSubtitles	143M	15.94

## Footnotes

1 More examples can be found in this Python package that collects recent word embeddings: https://github.com/plasticityai/magnitude
